# Supramolecular Arrangement of Doxycycline with Sulfobutylether-β-Cyclodextrin: Impact on Nanostructuration with Chitosan, Drug Degradation and Antimicrobial Potency

**DOI:** 10.3390/pharmaceutics15041285

**Published:** 2023-04-19

**Authors:** Renata Carvalho Feitosa, Juliana Souza Ribeiro Costa, Marcelo van Vliet Lima, Elina Sawa Akioka Ishikawa, Karina Cogo Müller, Fernando Bonin Okasaki, Edvaldo Sabadini, Claudia Garnero, Marcela Raquel Longhi, Vladimir Lavayen, Arnóbio Antônio da Silva-Júnior, Laura Oliveira-Nascimento

**Affiliations:** 1 Faculty of Pharmaceutical Sciences, University of Campinas (UNICAMP), Campinas 13083-871, SP, Brazil; 2 Department of Physical Chemistry, Institute of Chemistry, University of Campinas (UNICAMP), Campinas 13083-970, SP, Brazil; 3 Research and Pharmaceutical Technology Development Unit (UNITEFA, CONICET-UNC) and Department of Pharmacy, Faculty of Chemical Sciences, National University of Cordoba, Cordoba X5000HUA, Argentina; 4 Department of Inorganic Chemistry, Institute of Chemistry, Federal University of Rio Grande do Sul (UFRGS), Porto Alegre 91501-970, RS, Brazil; 5 Laboratory of Pharmaceutical Technology and Biotechnology, Department of Pharmacy, Federal University of Rio Grande do Norte (UFRN), Natal 59012-570, RN, Brazil

**Keywords:** cyclodextrin, chitosan, doxycycline, inclusion complex, nanoparticle, polysaccharide

## Abstract

Doxycycline (DX) is a well-established and broad-spectrum antimicrobial drug. However, DX has drawbacks, such as physicochemical instability in aqueous media and bacterial resistance. The inclusion of drugs in cyclodextrin complexes and their loading into nanocarriers can overcome these limitations. Thus, we studied the DX/sulfobutylether-β-CD (SBE-β-CD) inclusion complex for the first time and used it to reticulate chitosan. The resulting particles were evaluated by their physicochemical characteristics and antibacterial activity. DX/SBE-β-CD complexes were characterized by nuclear magnetic resonance, infrared spectroscopy, thermal analysis, X-ray diffraction, and scanning electron microscopy (SEM), whereas DX-loaded nanoparticles were characterized by dynamic light scattering, SEM, and drug content. The partial inclusion of the DX molecule in CD happened in a 1:1 proportion and brought increased stability to solid DX upon thermal degradation. Chitosan-complex nanoparticles measured approximately 200 nm, with a narrow polydispersity and particles with sufficient drug encapsulation for microbiological studies. Both formulations preserved the antimicrobial activity of DX against *Staphylococcus aureus,* whereas DX/SBE-β-CD inclusion complexes were also active against *Klebsiella pneumoniae*, indicating the potential use of these formulations as drug delivery systems to treat local infections.

## 1. Introduction

Doxycycline (DX) is a well-established antimicrobial drug that presents a broad-spectrum action. Its therapeutic applications encompass treatment and prophylaxis of several pathologies, which happen by administration through local or parenteral routes. Since this drug also inhibits matrix metalloproteinases (MMPs), its benefits are extended to the treatment of chronic inflammatory diseases and exacerbated inflammation caused by infections. However, some limitations hamper the use of DX, such as instability in aqueous media, bacterial resistance, and poor cellular penetration. In turn, nanostructured drug delivery systems can increase DX protection in the physiological environment, reduce side effects, enhance targeting, and disrupt antimicrobial resistance mechanisms [1].

Among the strategies for drug structuration in the nanometer range, cyclodextrins (CDs) form supramolecular structures by including guest molecules in their internal hydrophobic cavity. DX inclusion complexes were reported with hydroxypropyl-β-cyclodextrin (HP-β-CD) (non-ionic residues) and magnesium (chelation), which stabilized the drug and maintained the antimicrobial activity [2,3,4]. Other studies using β-CD indicated an increase in DX photostability [5] and a decrease in osteoblast cytotoxicity as a consequence of drug complexation [6]. However, there are no reports of inclusion complexes of DX with sulfobutylether-β-cyclodextrin (SBE-β-CD); this CD variant has enhanced water solubility, better drug binding capacity, low toxicity profile, and enhanced hemocompatibility when compared with its parental β-CD [7]. When comparing SBE with HP modifications of cyclodextrin, SBE moieties allow ionic interactions that may facilitate complexation with charged molecules.

Another valuable structuration strategy concerns chitosan-based nanoparticles (NPs). Chitosan is a polysaccharide extracted from seafood industry waste that offers mucoadhesion, low toxicity, and adjustable physical properties [8]. The most common method to prepare chitosan NPs is through ionic gelation with anionic molecules such as sodium tripolyphosphate (STPP). DX-loaded chitosan NPs were described with STPP [9,10,11] but also with triphenyl phosphate [12] and sodium alginate [13,14]. None of these papers intended pulmonary delivery. Noteworthily, these studies presented DX quantification by UV–Vis spectrophotometry, which precludes evaluation of degradation products detected at the same wavelength.

Polyphosphates belong to the substrate group for alkaline phosphatases (AP), which are present in mucus membranes and other body compartments. Due to this susceptibility, STTP–chitosan NPs released 90% of the drug payload after 15 min of contact with diluted intestinal AP in vitro, as opposed to 30% in the same timeframe in the absence of the enzyme [15]. The rapid leakage is probably favored by the predominant superficial crosslinks offered by STTP [16]. Considering lung infections that cause inflammation, AP has increased activity in the pulmonary fluid [17], which makes polyphosphates inadequate crosslinkers for local sustained release in the lungs. Concerning alginate reticulation, one study used a low amount of doxycycline (16 µg/mL), whereas the other demanded high-energy mixing [13,14].

As an alternative crosslinker, SBE-β-CD has 6–7 anionic charges that can reticulate chitosan to form nanoparticles [18,19,20,21]. These charges are permanent over the physiological range and not susceptible to enzymatic hydrolysis or other metabolization processes [22]. In addition, SBE-β-CD does not require dilution in specific pHs, such as alginate, nor need high energy to form nanoparticles. 

Based on the abovementioned information, our hypothesis was that DX would complex with SBE-β-CD, which could then crosslink with chitosan and that both would provide low nanosized polydispersity, drug incorporation, and equivalent antimicrobial potency. Therefore, physicochemical studies were performed to evaluate interactions, stability, and drug content, whereas in vitro antimicrobial evaluation was used to determine the drug pharmacological action. We believe our studies have provided a wide picture of the feasibility, benefits, and limitations of the explored strategies.

## 2. Materials and Methods

### 2.1. Material

Low-molecular-weight chitosan (viscosity: 20–300 cps, 75–85% deacetylated, Lot: SLBG1673V) was purchased from Sigma-Aldrich (São Paulo, Brazil). We had assessed in a previous study the molecular mass (4.61 × 10^5^ g mol^−1^) by the Mark–Houwink–Sakurada equation using the viscosimetric method and degree of deacetylation (88.16%) using the conductimetric method [19]. Doxycycline hyclate was purchased from Infinity Pharma (Campinas, Brazil). Sulfobutylether-β-cyclodextrin (Dexolve™) (MW: 2163.3 g mol^−1^, Degree of substitution: 6.5, Lot: CYL-4283) was donated from CycloLab (Budapest, Hungary). Ammonium oxalate, dimethylformamide, ammonium phosphate dibasic, hydrochloric acid, sodium hydroxide, ethanol, acetate and phosphate buffers, deuterium oxide, and phosphomolybdic acid were of analytical grade. The bacterial strains used were *Staphylococcus aureus* (ATCC 29213) and *Klebsiella pneumoniae* (ATCC BAA1705), both cultured in Mueller Hinton broth (Sigma-Aldrich). The purified water (1.3 µS cm ^−1^) was prepared from reverse osmosis purification equipment (model OS50 LX, Gehaka, Sao Paulo, Brazil).

### 2.2. Drug Content and Impurities by High-Performance Liquid Chromatography (HPLC) 

DX was quantified by a method adapted from the one described by Zhang et al. [4]. The analytical curve was performed in purified water from 5 to 100 μg/mL. The method performance data are described in the supplementary material (Tables S1–S3 and Figure S1). The HPLC system (Hitachi LaChrom Elite—Merck Hitachi, Tokyo, Japan) included a 250 mm × 4.6 mm (5 µ) Hypersil™ ODS C18 column (Thermo Fisher Scientific™, Waltham, MA, USA) maintained at 35 °C; mobile phase of 0.05 M ammonium oxalate, dimethylformamide, and 0.2 M ammonium phosphate dibasic (65:30:5, pH 8.0 ± 0.2); flow rate of 1.0 mL/min; and UV detection (280 nm). Impurities, which include degradation products, were calculated by the normalization procedure as described in the USP General Chapter 621: the area of a peak as a percentage of the total area of all the peaks, excluding those due to solvents, reagents, mobile phase, or the sample matrix and those at or below the limit at which they can be disregarded. The peak areas of impurities were summed up since we did not identify each individual peak.

### 2.3. DX/SBE-β-CD Complex Preparation

The complex was prepared by solubilizing DX and SBE-β-CD in purified water at a 1:1 or 1:4 molar ratio (DX: SBE-β-CD) and adjusting the pH to 5.0 with 0.1 M sodium hydroxide. Then, it was stirred for 24 h at 350 rpm (magnetic) and 25 °C, protected from light. The resultant complex solution was stored at 2–8 °C or freeze-dried. The Lyostar 3 (SP scientific) was used for sample lyophilization; the formulations were frozen in type I glass vials with rubber stoppers at −30 °C for 5 h, followed by vacuum (100 mTorr) and primary drying at −30 °C for 24 h and secondary drying at −20 °C for 16 h and 20 °C for 20 h.

### 2.4. Stability of DX/SBE-β-CD Complex in Different pHs

The drug complex solution (1:1) was diluted to 0.1 mg/mL of DX with: 0.1 N HCl (pH = 2), acetate buffer (pH = 5), phosphate buffer (pH = 8), and 0.5 N NaOH (pH = 12). After the initial analysis of drug content and impurities (0 h), the samples were kept under stirring (350 rpm, 25 °C, dark) for 24 h to perform the analysis. Free DX solution was submitted to the same conditions for comparison. This study was performed with a sample for each condition. 

### 2.5. Job’s Plot

The stoichiometry of complexes was evaluated by the continuous variation method (Job’s method) [23] and further confirmed by ITC. Solutions of DX and SBE-β-CD were prepared at 0.06 mM in acetate buffer (pH 5.0) and mixed at different volume ratios. The samples were analyzed by UV/Vis spectroscopy (Thermo Fisher Scientific, 60S Evolution, USA) at 275 nm and the difference in absorbance between the solutions with and without SBE-β-CD was related to “R” (Δabs × R), calculated by Equation (1), with substances in mM:

(1) $$R=\frac{\left[DX\right]}{\left[DX\right]+[SBE-\beta -CD]}$$

### 2.6. Isothermal Titration Calorimetry (ITC)

ITC experiments were conducted on a MicroCal Peaq^®^-ITC (Malvern, UK) at 25 °C. A 4.0 mmol/L solution of SBE-β-CD was titrated with a 35 mmol/L solution of DX, both dissolved in a 10 wt.% solution of ethanol in water, pH 5.0. The titrations were carried out with 1 injection of 0.5 µL (heat discarded) and 18 subsequent injections of 2.0 µL of the titrant spaced with 600 s between one another. The stir speed was set to 750 rpm, and the reference power was set to 7.0 µW without feedback. Control experiments were conducted with DX solution titrated in the 10 wt.% ethanol–water solution at pH 5.0 without SBE-β-CD, and the correspondent heat of dilution of DX was discounted during the heat of binding analysis. All titrations were made in duplicate. The obtained calorimetric data was analyzed with “MicroCal PEAQ-ITC Analysis Software”, and the enthalpogram was fitted to the “one set of sites” model to obtain the thermodynamic parameters of the binding.

### 2.7. Proton Nuclear Magnetic Resonance (1H NMR) and 2D-NOESY Spectra

Freeze-dried samples were solubilized in deuterium oxide (D_2_O) to obtain a final concentration of 30 mg/mL. Each solution was transferred to 5 mm tubes and hermetically sealed. The 1H NMR and 2D-NOESY sample spectra were obtained on a Bruker Advance spectrometer of 500 MHz.

### 2.8. Fourier Transform Infrared Spectroscopy (FTIR)

Fourier transform infrared spectroscopy analyses using the attenuated total reflection accessory (FTIR-ATR) were performed for the 1:4 molar ratio complex in a Shimadzu^®^ IR-Prestige-21 (Tokyo, Japan), the MID (middle infrared) spectral region ranging from 700 to 4000 cm^−1^, with a resolution of 4.0 cm^−1^ and 20 scans for each analysis. The analyses for the complex at a 1:1 molar ratio was performed in a Thermo Scientific^®^ Nicolet 6700 (Madison, WI, USA), the MID spectral region ranged from 675 to 4000 cm^−1^, with a resolution of 4.0 cm^−1^ and 128 scans for each analysis.

### 2.9. Thermogravimetric Analysis (TGA) and Differential Scanning Calorimetry (DSC)

The TGA and DSC (perforated alumina pans) analyses were carried out using a thermogravimetric analyzer (Mettler Toledo, model TGA/DSC1, Schwerzenbach, Switzerland). The analyses were performed in nitrogen atmosphere at a flow rate of 50 mL/min, with samples heated from 25 to 300 °C at a rate of 10 °C per minute.

### 2.10. X-ray Diffraction (XRD)

Solid samples were analyzed using an X’Pert-MPD X-ray diffractometer (Philips Analytical X-ray) equipped with a source of Cu Kα X-ray radiation (λ = 1.54056 Å) (Almelo, The Netherlands). The samples were transferred to a glass sample holder (cavity of 10 mm × ~0.6 mm) and manually pressed with a glass slide. The equipment was set at 40 kV, 40 mA, 2-theta scan range of 5–50°, step size of 0.050° and scan speed of 0.033°/s.

### 2.11. Hydrodynamic Diameter and Zeta Potential Measurements

The mean particle size (PS) of samples were evaluated by nanoparticle tracking analysis (NTA, NanoSight NS300, Malvern, UK) at 25 °C and ultrapure water dilution to obtain 30–100 particles per frame and 10^7^–10^9^ particles per mL. Mean hydrodynamic diameter (particle size, PS), polydispersity index (PDI), and zeta potential (ZP) of samples were determined using a Zetasizer Nano ZS (Malvern Instruments, Malvern, UK). DLS analyses were performed at 173°, 25 °C, and dilution in ultrapure water at 1:10 (*v*/*v*) (refractive index 1.333—viscosity 0.8905 cP). The measurements were performed for at least ten determinations for each sample, in triplicate, and data were expressed as mean ± standard deviation.

### 2.12. Scanning Electron Microscopy (SEM)

Solid samples (free DX, SBE-β-CD, physical mixture, and complex) were observed using a scanning electron microscope (LEO 440i) with an X-ray dispersive energy detector 6070 (LEO Electron Microscopy, England). These samples were placed on a glass slide and fixed to the stub with double-sided carbon tape. Afterwards, metallic coating with gold (200 A°) was performed using K450 Sputter Coater EMITECH (Kent, Reino Unido). Due to the nature of the sample, the preparation of nanoparticles (liquid dispersion) for SEM analysis was performed differently from that performed for solid samples. To improve the visualization of the particles and ensure their stability during the vacuum drying step, phosphomolybdic acid was used. Field emission gun scanning electronic microscopy (Zeiss Microscopy, Auriga, Jena, Germany) images were used to evaluate nanoparticle morphology: one drop of each sample (liquid dispersion of empty or DX-loaded nanoparticles) was put on the carbon tape slide followed by one drop of 1% *w*/*v* phosphomolybdic acid. The samples were kept in a desiccator for 24 h at room temperature before the analysis. The change in equipment for analysis of liquid and solid samples happened due to the availability of analysis and not because of sample requirements.

### 2.13. Chitosan Nanoparticles Formulation

Chitosan (CS) nanoparticles were prepared by ionic gelation method between CS and SBE-β-CD [24]. The nanoparticle formation was detected by varying CS and SBE-β-CD concentrations. Briefly, 1 mL of SBE-β-CD aqueous solution was added dropwise onto 3 mL of CS solution (diluent 1% (*v*/*v*) acetic acid, pH 5.0, filtered with a 0.45 μm PVDF filter) under magnetic stirring (350 rpm) at room temperature for 30 min (NPs). The formulation with the best parameters for average size and polydispersity index (PDI) was chosen for the incorporation of DX complexes (described in Section 2.2), with NPs prepared as described for the empty ones.

### 2.14. Drug Content and Encapsulation Efficiency in NPs

DX-loaded nanoparticles (750 µL) were mixed and vortexed with HCl 0.1 M (750 µL) and centrifuged for 30 min at 14.000× *g*, 25 °C. The supernatant was filtered (0.45 μM) and the DX content was quantified by HPLC as described in Section 2.3. The percentage of encapsulated drug in the nanoparticles was determined using the indirect method of filtration–centrifugation. Nanoparticles were centrifuged (Centrifuge 5810R, Eppendorf, Germany) for 40 min at 14.000× *g*, 25 °C, in 0.5 mL ultrafiltration devices (Amicon^®^ Ultra 30 kDa, Millipore, Germany). The encapsulation efficiency (EE) was calculated by Equation (2), where [DX]_total_ is the total drug content and [DX]_free_ is the drug quantified in filtrate after centrifugation. The measurements were performed in triplicate and data were expressed as mean ± standard deviation.

(2) $$EE(\%)=\frac{{\left[DX\right]}_{total}-{\left[DX\right]}_{free}}{[{DX]}_{total}}\times 100$$

### 2.15. Minimum Inhibitory Concentration (MIC)

The samples were evaluated by the microdilution method on 96-well plates as described by the Clinical and Laboratory Standards Institute (CLSI) [25]. The bacterial strains tested were *Staphylococcus aureus* ATCC 29213 and *Klebsiella pneumoniae* ATCC BAA1705, both cultured in Mueller Hinton broth. After serial dilutions of the samples and microbial addition, the plates were incubated for 24 h at 37 °C. Samples of free SBE-β-CD and empty nanoparticles were used as control groups. The MICs were calculated by the rise in optical density (550 nm) and confirmed with change in the color of resazurin (30 µL/well of 0.01% aqueous solution). All tests were performed in triplicate.

## 3. Results and Discussion

### 3.1. Characterization of DX/SBE-β-CD Complexes in Aqueous Solution

#### 3.1.1. The Stability of the DX/SBE-β-CD Complex in Different pHs

We first studied the stability of DX/SBE-β-CD at different pHs, since the antibiotic degrades in basic solutions [26,27]. Drug recovery declined considerably at alkaline pH, and even further after 24 h, for both complex and free drug; as expected in acidic pH, the drug was recovered at high rates, presenting no degradation in pH 5 (Table 1). This behavior has been previously reported for free DX in aqueous solution [28,29]. The conversion of DX into its enol form (keto-enol tautomerism) is favored in basic medium and may be related to its stability [28,30]. The molecule tends to epimerize, forming 4-epidoxycycline, in acidic medium (below 5.0) [31]. 

The main impurities of DX raw material are 6-epidoxycycline (6-EDX), metacycline (MTC), 4-epidoxycycline (4-EDX), 4-epi-6-epidoxycycline (4,6-EDX), oxytetracycline (OTC), and 2-acetyl-2-decarbamoyldoxycycline. DX is synthesized from OTC, therefore MTC and 6-EDX are intermediate products that can be present as synthetic impurities. The DX molecule can also epimerize into 4-EDX and 4,6-EDX during synthesis or depending on the storage conditions [32]. These epimerizations are reversible but have low antibacterial activity and some toxicity [33]. Although the DX/SBE-β-CD complex did not offer protection in solution (in the molar ratio, concentration, pH, and solvents tested), several studies that encapsulate DX in nanoparticles do not monitor samples by a stability-indicating method, so one cannot evaluate the contribution of degradation products in the physicochemical stability of these formulations. 

#### 3.1.2. DX/SBE-β-CD Complex Stoichiometry and Binding Studies

**Figure 1:**
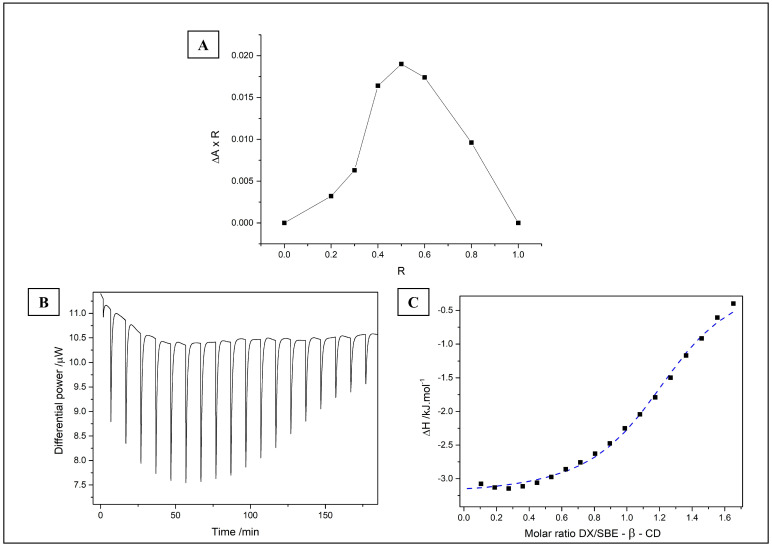
(**A**) Job’s plot of UV/Vis absorbance at different ratios (R) of doxycycline DX and SBE-β-CD in acetate buffer (pH 5.0). Delta A = delta absorbance between free DX and DX in complex form. (**B**) Raw calorimetric data of the titration of DX with SBE-β-CD. (**C**) Enthalpogram of the binding at pH 5.0/25 °C in 10 wt% solution of ethanol in water. Dashed blue line = fitting into the “one set of sites” model.

The complex formed in pH 5.0 due to DX stability and its zwitterion form at this pH. The zwitterion may interact electrostatically with anionic cyclodextrin molecules and engage in hydrophobic interactions. The stoichiometry of the complexes was evaluated by the continuous variation method (Job’s plot, Figure 1A). The maximum change in absorbance (ΔA × R) was observed at R = 0.5, which suggests the 1:1 stoichiometry [34]. The same proportion was reported for DX complexes with β-CD and γ-CD [35]. However, it is important to note that Job’s plot is a preliminary method that cannot be used solely to determine inclusion complex ratios [36].

Isothermal titration calorimetry (ITC) experiments give a thermodynamic view of the studied phenomenon and can confirm stoichiometry. Since SBE-β-CD is expected to have a single binding site for DX, analogous to what has been previously reported in the literature for DX with other CD derivatives [6], the enthalpogram was fitted to the “one set of sites” model to obtain the thermodynamic parameters of the binding.

Figure 1B shows the raw calorimetric data obtained in the titration of DX with SBE-β-CD, and the respective enthalpogram is in Figure 1C, normalized by the control experiment. The dashed line corresponds to the fitting in the “one set of sites” model, from which we calculated the thermodynamic parameters of the binding phenomenon. The binding is exothermic (ΔH = −3.2 ± 0.1 kJ mol^−1^), associated with the heat release during the binding that is related to the establishment of favorable interactions between DX and SBE-β-CD. The obtained enthalpy value agrees with the reported one of −3.8 kJ mol^−1^ for DX and β-cyclodextrin (β-CD) [6]. The positive value of TΔS (19.3 ± 0.8 kJ mol^−1^) also complies with what is reported in the literature with DX and β-CD, being −11.6 kJ mol^−1^, in which the increase in entropy relates to the release of solvation water in both DX and SBE-β-CD [6]. Overall, the ΔG of binding of DX and SBE-β-CD (ΔG = −22 ± 1 kJ mol^−1^) is larger than the reported value of −15.4 kJ mol^−1^ for DX and β-CD, indicating a more favored process. 

The obtained number of sites (*n* = 1.1 ± 0.1) agrees with the 1:1 stoichiometry reported for the binding of cyclodextrins [6,37] and with the Job’s plot result in this work. Concerning the affinity constant (Kc), a Kc > 1000 indicates a relatively strong binding system [6,38]. The obtained value of Kc (5917 mol^−1^ L) is an order of magnitude higher than that reported for DX and β-cyclodextrin (Kc = 503 mol^−1^ L) [6]. The larger Kc accompanies the larger ΔG, indicating that the binding of DX and SBE-β-CD is more favored when compared with the binding of DX and β-CD.

#### 3.1.3. 1H NMR and 2D-NOESY Spectra

**Figure 2:**
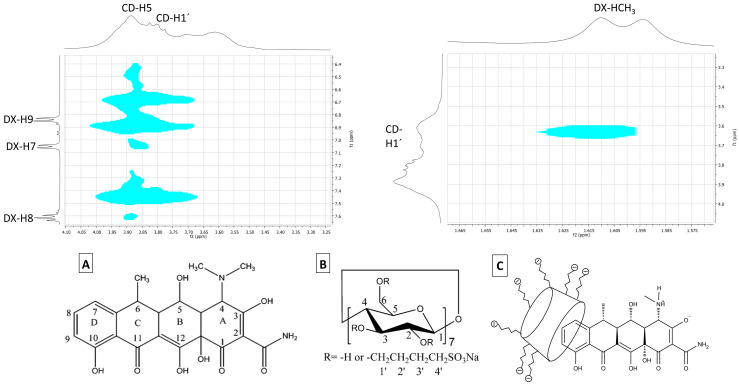
**Top**: contour plot of the 2D-NOESY spectra of DX/SBE-β-CD inclusion complex. DX: doxycycline. CD: sulfobutylether-β-cyclodextrin (SBE-β-CD). **Bottom**: (**A**) Molecular structure of doxycycline. (**B**) Molecular structure of SBE-β-CD. (**C**) Proposed geometric arrangement for the DX/SBE-β-CD inclusion complex.

The interactions between DX and SBE-β-CD molecules were investigated in the complex (1:1 molar ratio) dissolved in deuterated water solution. In the presence of SBE-β-CD, the DX proton resonances showed significant shifts to downfield. In contrast, slight displacements for protons of the macromolecule were observed (Table S4). The 2D-NOESY showed few weak points of interaction between the molecules. In the expanded contour map, cross peak correlations between aromatic DX protons (ring D) and the H5 (proton located in the hydrophobic cavity) of SBE-β-CD were observed, suggesting that the inclusion of a drug molecule in CD occurs on the aromatic side (Figure 2). The NMR spectra and molecular structures of the drug and cyclodextrin can be seen in detail in Figures S2 and S3.

Therefore, the diketone phenol group remains exposed and could facilitate the hydrolysis of the molecule [2], which could explain the low stability of DX in aqueous solutions demonstrated in Section 3.1.1. Another study showed that the drug molecule enters the same side in the DX/β-CD inclusion complex [39]. The amide and tertiary amine groups in the aliphatic group are hydrophilic and bulky, so they do not favor DX inclusion into that side [6].

#### 3.1.4. Hydrodynamic Diameter (Size)

The particle size distribution of DX/SBE-β-CD complexes was assessed by NTA. The results indicated a mean hydrodynamic diameter of 234.0 ± 8.7 nm, with D10 = 129.5 ± 10.9 nm, D50 = 212.8 ± 10.8 nm, and D90 = 369.3 ± 7.7 nm: Span = 1.1269. The sample concentration was 8.0 ± 0.2 × 10^8^ particles/mL. The particle size data for cyclodextrin complexes are scarce in the literature, especially for SBE-β-CD. DX/HP-β-cyclodextrin inclusion complexes were reported to present a mean diameter of approximately 245 nm (1:2 molar ratio, DLS measurements) [39], which is similar to our result. It is likely that part of cyclodextrin complexes aggregate and form supramolecular structures [40], indicated by the obtained D90 value, but still in the submicron size range.

### 3.2. Characterization of DX/SBE-β-CD Complexes in Solid State

#### 3.2.1. Fourier Transform Infrared Spectroscopy (FTIR)

**Figure 3:**
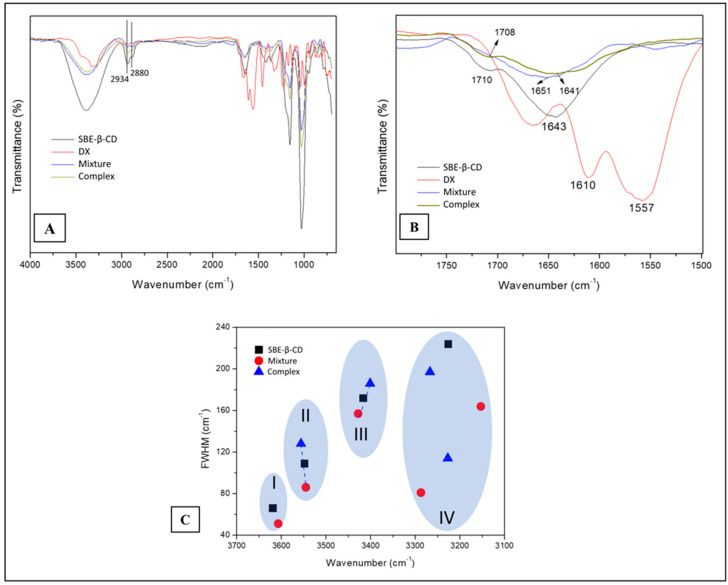
Infrared spectra from 4000 to 650 cm^−1^ (**A**) and 1800 to 1500 cm^−1^ (**B**) of samples of doxycycline (DX), sulfobutylether-β-cyclodextrin (SBE-β-CD), physical mixture, and freeze-dried complex (1:4 molar ratio). (**C**) Full width at half medium (FWHM) as a function of wavenumber in the range of 3700–3100 cm^−1^ of the samples. The shadow area is related to regions (I, II, III, IV) with a maximum wavenumber.

The analysis was carried out in a preliminary way using a 1:4 molar ratio (DX/SBE-β-CD) for complex and physical mixture. The spectra (Figure 3A) presented bands in all the samples at 2934 cm^−1^ and 2880 cm^−1^, related to stretching mode C-H (spectral assignments in Table S5). 

The spectral region of 1800–1500 cm^−1^ (Figure 3B) is associated with the host–guest interactions (bending H-O-H mode at 1664 cm^−1^). The respective bands of the physical mixture (1651 cm^−1^) and complex (1641 cm^−1^) presented similar intensities. The wide wavenumber band of the complex is higher than the mixture and relates to the formation of the solid-phase complex. The band at 1643 cm^−1^ for the SBE-β-CD pure sample is approximately two times more intense than the one for the inclusion compound. This region is sensitive to intracavity water; thus, we can conclude that DX is partially inside the CD cavity, which agrees with the NMR data.

We identified four spectroscopic signal regions using the deconvoluted bands between 3100 cm^−1^ and 3700 cm^−1^ (Figure 3C). The 3610 cm^−1^ region (I) relates to water antisymmetric (ν3) mode; the second region at 3515 cm^−1^ (II) relates to a cluster of intracavity water molecules located in a hydrophobic environment. The last two spectral signals were at 3410 cm^−1^ (III), related to H-bond presence into the cyclodextrin macrocycles, and the mode at 3330–3190 cm^−1^ (IV) associated with symmetric (ν1) OH stretching active mode. We observed two types of wavenumber shifts in the regions named II and III. A more favorable chemical environment for the existence of hydrogen bonds occurs at lower wavenumber values (region III). The value of FWHM shows an opposite tendency (regions II and III), indicating the presence of a breakdown of the intramolecular hydrogen bonding with their reorganization in the inclusion compound. Therefore, a dynamic chemical environment is present among the inclusion compound, physical mixture, and the SBE-β-CD samples. 

These spectral findings are supported by the observations at 1557 cm^−1^, 1610 cm^−1^, and 1651 cm^−1^, which are associated with the DX molecule’s s(C=C) active modes. The former band is absent in the inclusion complex spectrum, and the latter two bands reduced their intensity, suggesting that the guest DX ring could be inside the host cavity. As a comparison, reports on the complexes of 1,2-naphthalenes with β-cyclodextrin [41,42] showed that the 2-naphthalenes are included axially into the cyclodextrin cavity better than the 1-naphthalenes. In our case, SBE-β-CD has a carbonyl group on the 1-position of the ring, which can lead to a similar situation to the naphtalenes.

The weak intensity band at 1643 cm^−1^ (stretching C=O mode) relates to DX: its value, the highest wavenumber shift, is observed in the physical mixture and in the inclusion compound, which has a higher value than the reference’s one. It means that the inductive effect increments have a loss of resonance on the carbonyl, affecting the force constant of the C=O bond, which results in a higher wavenumber.

In the region from 1500 to 1200 cm^−1^, we identified bands at 1456 cm^−1^, 1420 cm^−1^, and 1366 cm^−1^, which are associated with deformations in the active modes of the C-H in the primary and secondary hydroxyl groups of the cyclodextrin molecule (Table S5). We also identified the main modes below 1000 cm^−1^ related to the supramolecular system’s host–guest. Thus, the bands at 946 cm^−1^, 858 cm^−1^, and 788 cm^−1^ are attributed to the ring molecule of the guest.

**Figure 4:**
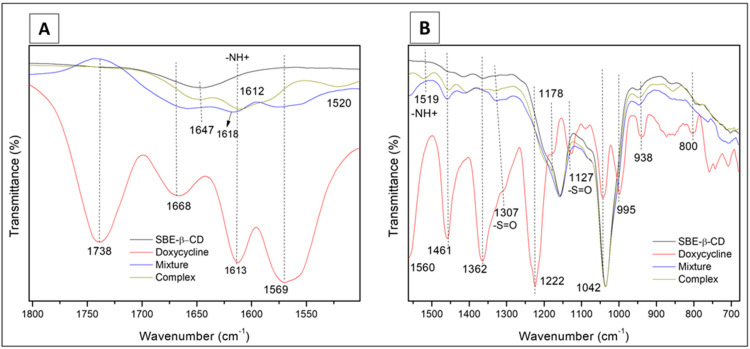
Infrared spectra from 1800 to 1500 cm^−1^ (**A**) and 1560 to 700 cm^−1^ (**B**) of samples of doxycycline (DX), sulfobutylether-β-cyclodextrin (SBE-β-CD), physical mixture, and freeze-dried complex (1:1 molar ratio).

**Figure 5:**
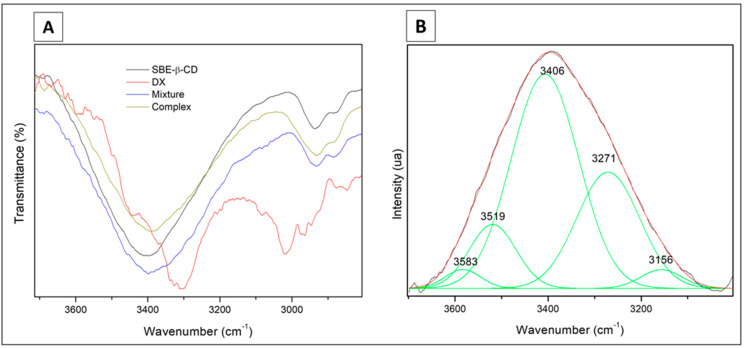
Infrared spectra from 3700 to 3000 cm^−1^ plotted as transmittance vs. wavenumber (**A**) and intensity vs. wavenumber (**B**) for samples of doxycycline (DX), sulfobutylether-β-cyclodextrin (SBE-β-CD), physical mixture, and freeze-dried complex (1:1 molar ratio).

When we analyzed the differences between the 1:1 (Figure 4) and 1:4 molar ratios of the freeze-dried complex, we identified five signals in the range 3700–3000 cm^−1^ for the 1:1 molar ratio complex (Figure 5). The four region (I, II, III, IV) signals correspond to 3519 cm^−1^, 3406 cm^−1^, 3271 cm^−1^, and 3156 cm^−1^, respectively. The fifth one at 3583 cm^−1^ is associated with the N-H weak interactions of the guest molecule. The signals of the four regions (I, II, III, IV) between the 1:1 and the 1:4 inclusion compounds have slight differences due to the dynamic organization bond. However, it corroborates with the conclusions of the 1:4 analysis concerning the complex structuration. 

#### 3.2.2. Thermal Analysis

**Figure 6:**
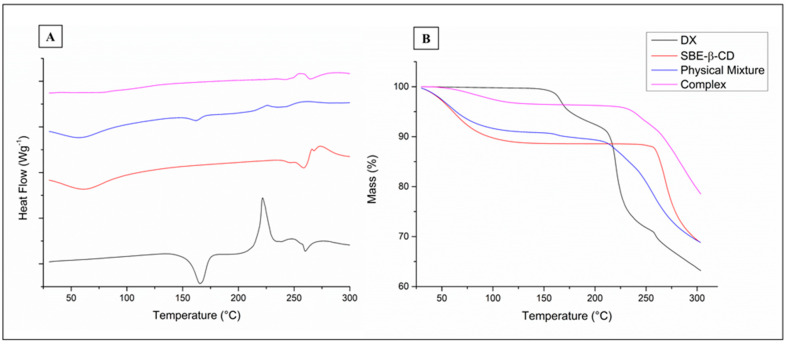
DSC (**A**) and TGA (**B**) thermograms of doxycycline, sulfobutylether-β-cyclodextrin (SBE-β-CD), physical mixture (1:1), and the DX/SBE-β-CD (1:1) complex.

DSC data (Figure 6A) for DX shows an endothermic peak at 165.6 °C and an exothermic peak at 221.6 °C (onset of 202 °C). The TGA data (Figure 6B) for DX presents two weight loss events (160 °C and 216 °C) corresponding to the mentioned DSC peaks. Then, a following DX treatment up to 170°C was analyzed by HPLC and revealed no increase in related substances, which excludes DX degradation as the reason for the first mass loss. This loss percentage is similar to the mass percentage of solvents in doxycycline hyclate (6.6%) and happens at a temperature range possibly related to water/ethanol evaporation in complex mixtures. We then assumed that the endothermic peak was a consequence of solvent evaporation. The second peak was attributed to doxycycline carbonization since a manufacturer confirms that DX chars without melting at 201 °C [43]. 

The mass loss observed for DOX and the physical mixture between 120 and 225 °C was not present in the inclusion complex analysis. The SBE-β-CD graph of DSC shows a broad endothermic peak around 61 °C (dehydration), with decomposition (approximately 260 °C) confirmed by the weight loss in the TGA curve and by previous reports [24]. The DSC curve of the physical mixture (DX and SBE-β-CD) shows the same peaks registered for the individual compounds, whereas the inclusion complex graph does not have the peak attributed to solvent evaporation of DX; the same phenomenon was reported for complexes of DX/β-CD [5]. 

The TGA curves demonstrate that thermal degradation occurred at higher temperatures for the inclusion complex, which indicates a higher resistance to heat treatment. Since previous studies showed that DX powder, tablets, and pulverized tablets of DX degraded in accelerated conditions (40 °C and 75% of humidity for 3 months), the inclusion complexes studied here can be used in solid formulations to improve the drug thermal stability [44]. 

#### 3.2.3. X-ray Diffraction (XRD)

**Figure 7:**
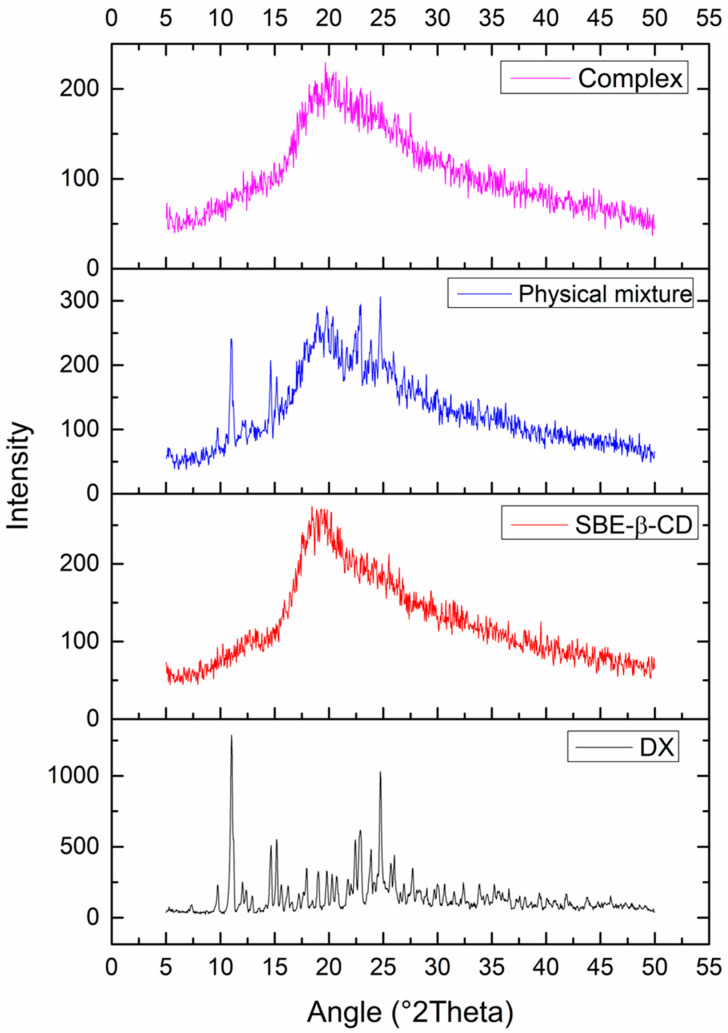
X-ray diffraction patterns of the DX/SBE-β-CD (1:1) complex, physical mixture (1:1), sulfobutylether-β-cyclodextrin (SBE-β-CD), and doxycycline (DX).

This assay was performed to investigate the physical form (crystalline or amorphous) of the drug, cyclodextrin, physical mixture, and complex (Figure 7). XRD can also be used to indicate the formation of inclusion complexes, since complexation leads to drug amorphization and consequent decrease or absence of crystalline peaks in the diffractogram. The DX diffractogram shows characteristic sharp peaks (2θ: 11.06, 14.65, 15.16, 22.44, 22.94, 24.78) that are present in the physical mixture at minor intensities due to drug dilution with cyclodextrin. As expected, the diffractogram of the complex showed a drastic reduction in the crystallinity index, suggesting the formation of an inclusion complex and corroborating with previous tests.

#### 3.2.4. Scanning Electron Microscopy (SEM)

**Figure 8:**
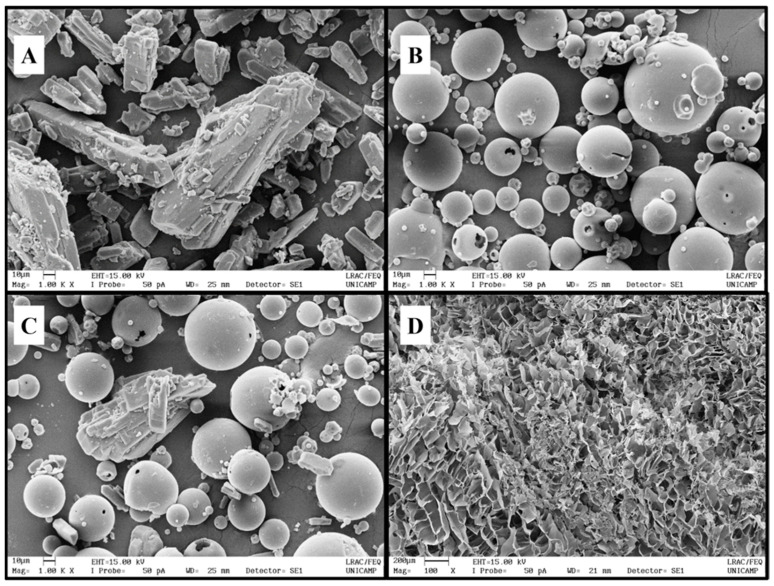
Scanning electron microscopy of (**A**) doxycycline hyclate (DX), (**B**) sulfobutylether-β-cyclodextrin (SBE-β-CD), (**C**) the physical mixture, and (**D**) the DX/SBE-β-CD complex.

SEM analysis was performed to evaluate the morphology of the DX/SBE-β-CD inclusion complex. DX (Figure 8A) appeared as a monoclinic crystal system [45], whereas SBE-β-CD (Figure 8B) exhibited spherical particles. In the physical mixture sample (Figure 8C), a blend of DX crystals and SBE-β-CD particles can be visualized. Lastly, the inclusion complex (Figure 8D) presented an irregular form and porous aspect (due to the freeze-drying process), which was quite different from raw materials of pure compounds, suggesting the formation of an inclusion complex.

### 3.3. Development and Characterization of Empty and DX-Loaded Nanoparticles

The molar ratio between CS and SBE-β-CD is critical for nanoparticle formation and particle size. Based on previous studies [24], different concentrations of CS and SBE-β-CD were tested, whereas the volume ratio was kept constant at 3:1 (CS:SBE-β-CD). The systems obtained were classified as “clear solution”, “opalescent dispersion”, or “aggregates” depending on the visual analysis and particle size. The polydispersity index (PDI) and zeta potential were determined for samples probably containing nanoparticles (Table 2). 

Initial tests considered 0.2 mg/mL of CS and variations from 0.8 to 3.0 mg/mL of SBE-β-CD (samples “A” to “D”). Increasing concentrations of the anionic reticulant led to the formation of aggregates. According to previous studies, the increased shielding of chitosan’s positive charge (observed by zeta potential decrease) leads to a reduction in repulsive forces between the NPs and formation of cross-bridges between the particles, resulting in agglomeration [24,46]. For the samples with a mass of crosslinking agent lower than the CS mass, NPs may have assembled in small amounts or may not have assembled at all, as probably occurred in sample “A” (clear to opalescent dispersion). The opalescent dispersion formed upon 0.2 mg/mL of CS and 1.2 mg/mL of SBE-β-CD (sample “B”) had the lowest values for size and PDI, indicating the formation of appropriate monodispersed nanoparticles.

A CS concentration of 0.2 mg/mL is low compared with other formulations of DX-loaded NPs reported in the literature (0.5 to 2.0 mg/mL of CS), so the concentration of 0.5 mg/mL was also tested to obtain a more concentrated formulation that was probably capable of a higher drug loading [9,10,12]. CS:SBE-β-CD mass ratio of approximately 1:1 and 1:2 worked better in the 0.2 mg test and were repeated for the 0.5 mg test. However, sample “F” showed aggregates, whereas sample “E” resulted in a particle size twice as large as sample “B”. Considering that reduced particle size and monodispersity may favor drug targeting [47,48], sample “B” was selected for drug incorporation.

**Figure 9:**
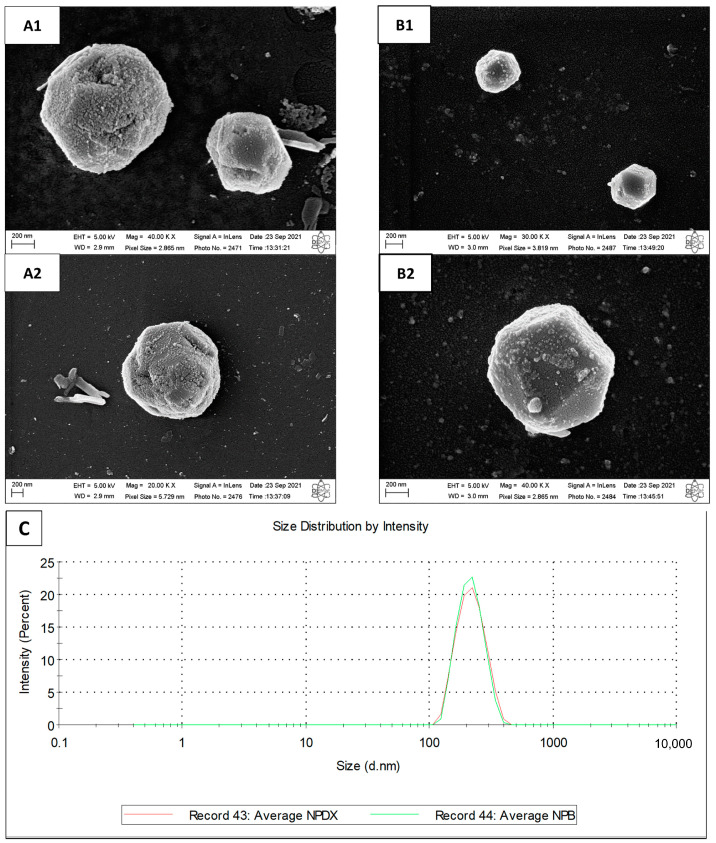
Scanning electron microscopy of (**A1**,**A2**) empty nanoparticles and (**B1**,**B2**) doxycycline-loaded nanoparticles. (**C**) Particle size distribution by percentage of intensity for empty nanoparticles (NPB) and doxycycline-loaded nanoparticles (NPDX).

DX-loaded nanoparticles presented a size of 209.7 ± 4.9 nm (Figure 9C), PDI of 0.039 ± 0.017, zeta potential of +17.5 ± 1.1 mV, and encapsulation efficiency of 25.19 ± 2.61%. The final pH of formulation was 5.11 ± 0.09. Nanoparticle morphology was assessed by SEM (Figure 9A,B). The images show polyhedral or spherical particles with irregular and rough surfaces and particle sizes according to DLS results. Mahmoud et al. [24] and Zhao et al. [19] also prepared chitosan/SBE-β-CD NPs and visualized particles with irregular edges. It is possible that the vacuum-drying step caused the particles to wither, resulting in jagged edges.

The amount of DX added in NP formulation (final concentration = 61.5 µg/mL) corresponds to the molar ratio DX:SBE-β-CD of 1:1. Thus, we did not evaluate the influence of drug concentration variation on encapsulation efficiency. As mentioned before, there are no reports of DX-loaded chitosan NPs using SBE-β-CD as the crosslinker. However, among the studies that use STPP [9,10,11], the mass of DX added to the formulation was much higher than the mass of chitosan, which may indicate that saturation of the polymer matrix could be necessary for satisfactory encapsulation (EE = ~56% and ~78%). In addition, the pH of the chitosan solution in acetic acid was adjusted to values between 5.5 and 6.0, which can also influence the EE due to the ionization rate of molecule. DX tends to lose the cationic group and gain a new anionic group as the pH of the medium becomes more alkaline. Under these conditions, the drug molecule could interact with the chitosan molecule through electrostatic interactions.

The NMR study indicates that inclusion of the DX molecule in SBE-β-CD occurs on the aromatic side. Therefore, functional groups that are ionized positively (NH_3_^+^) and negatively (CO-) remain exposed to the dispersion medium, interacting with other formulation components. It is likely that the positive charges of chitosan repel the cationic drug, hindering its encapsulation.

Although the Job’s plot and ITC indicated a 1:1 complexation stoichiometry, it is possible that an excess of CD is necessary for the complex structure to be maintained, since most of the interactions occur by weak Van der Waals forces. Couto et al. showed that to maintain the complexation with a 1:1 stoichiometry of capsaicin/HP-β-CD, 4.2 moles of CD were needed for each molecule of the active ingredient [37]. A compilation of studies that prepared CD complexes with different drugs also indicated the need for a surplus amount of CD [49]. Therefore, one might infer that the amount of CD was not enough to maintain the inclusion complex structure in this study, which may have led the hydrophilic DX molecules to stay dispersed in the medium rather than forming complexes. This behavior may also be associated with the difficulty of encapsulating the complex in NPs. We did not perform FTIR with this formulation because of the superposition of peaks. 

### 3.4. Antimicrobial Activity

The minimal inhibitory concentration (MIC) assay was performed to evaluate the antimicrobial activity of free DX, the DX/SBE-β-CD inclusion complex, and DX-loaded nanoparticles against Gram-positive and Gram-negative bacteria. DX inhibited *Staphylococcus aureus* (Gram-positive, ATCC 29213) proliferation up to a dilution of 0.5 µg/mL, regardless of the formulation with complex, NPDX, or in solution. Inhibition of *Klebsiella pneumoniae* (Gram-negative, ATCC BAA1705) needed 31.25 µg/mL (as it is a resistant strain) for free DX and complexed antibiotic. NPDX values could not be determined because its maximum concentration on the plate (31.25 µg/mL) was not inhibitory.

Reports [50,51,52] indicate different values of MIC for free DX against *S. aureus* ATCC 29213, ranging from 0.12 µg/mL to approximately 32 µg/mL, which agrees with our results. Suárez et al. [6] reported that DX inclusion complexes with β-cyclodextrin (β-CD) (MIC < 0.009 µg/mL) were more active against *S. aureus* than free DX (MIC = 1.22 µg/mL) was; however, this was with a different strain (ATCC 27664) from the one used in our study. The authors suggested that the reduced MIC value for the inclusion complex must be due to adhesion of β-CD to the bacterial surface by hydrogen bonds, which leads to a synergistic effect with the drug. NPDX formulation has the advantage of having positive zeta potential (+17.5 mV), which could facilitate adhesion to bacterial surface. However, considering that DX encapsulation efficiency was about 25%, most of the drug is free in the medium, leading to similar results among free drug, complex, and NPDX samples.

In relation to *K. pneumoniae* ATCC BAA-1705 (carbapenemase producer), free DX and complex samples showed the same MIC against this strain, but the MIC for NPDX formulation was not reached. Since the highest concentration tested (31.25 µg/mL) is the MIC value for this strain, the encapsulated amount of DX may not have been completely released within the first hours of incubation, resulting in antimicrobial growth. Higher drug concentrations were not tested because of the doxycycline content inside NPs.

## 4. Conclusions

In this study, inclusion complexes of DX/SBE-β-CD and respective DX-loaded nanoparticles were developed and characterized by physicochemical and morphological analyses for the first time. The resulting inclusion complexes presented a 1:1 molar ratio and their occurrence was confirmed by shifts in thermal profiles, drug amorphization (XRD), and changes in morphology (SEM). Complexation was able to enhance the thermal stability of DX in the solid state but did not improve its degradation profile in solution at room temperature (25 °C). NMR indicates that the inclusion of the DX molecule in CD occurs on the aromatic side, thus part of the molecule remains exposed to the aqueous medium and hydrolysis can occur on the opposite side. DX’s antimicrobial activity was maintained when complexed with SBE-β-CD against strains of *S. aureus* and *K. pneumoniae*. Therefore, the novel complex described may aid novel solid formulations with increased thermal resistance and preserved antimicrobial action.

Nanoparticles (NPDX) were obtained by reticulation of chitosan with the drug complex, with narrow size distribution and positive zeta potential, which aids in colloidal stability. Encapsulation efficiency was not high as expected (25%) but enough to proceed to biological evaluation. NPDX was as effective as DX against *S. aureus* but not against *K. pneumoniae*, which may be related to the release kinetics in culture media. Although successful particle parameters were obtained, the low encapsulation requires further studies with process and formulation design for NPDX to stand as a viable option for DX delivery.

## Figures and Tables

**Table 1:** Doxycycline (DX) and impurity (DX-IP) content in solutions of pure (free drug) and complexed drug (Complex) at different pH values.

pH	DX Complex (%)		DX Free Drug (%)		DX-IP Complex (%)		DX-IP Free Drug (%)	
	0 h	24 h	0 h	24 h	0 h	24 h	0 h	24 h
2	95.33	95.76	96.44	93.15	1.64	1.63	1.61	1.63
5	102.11	100.39	100.17	98.70	1.52	2.09	1.55	2.07
8	95.87	76.75	91.01	75.05	1.61	8.12	1.63	8.43
11	91.09	68.86	89.45	69.17	4.86	25.35	4.40	24.63

**Table 2:** Formulation parameters and visual analysis results of initial screening tests.

Sample	CS (mg/mL)	SBE-β-CD (mg/mL)	CS/SBE-β-CD Mass Ratio	Visual Analysis	PS (nm)	PDI	ZP (mV)
A	0.2	0.8	1:1.3	Clear to opalescent dispersion	523.0 ± 18.5	0.353 ± 0.073	+41.1 ± 1.5
B	0.2	1.2	1:2	Opalescent dispersion	208.8 ± 4.4	0.020 ± 0.018	+18.7 ± 1.4
C	0.2	2.0	1:3.3	Aggregates	-	-	-
D	0.2	3.0	1:5	Aggregates	-	-	-
E	0.5	1.8	1:1.2	Opalescent dispersion	438.9 ± 4.9	0.276 ± 0.020	+44.3 ± 1.0
F	0.5	3.0	1:2	Aggregates	-	-	-

“-“: not evaluated; CS: chitosan; PS: particle size; PDI: polydispersity index. The results are expressed as mean ± standard deviation (*n* = 3).

## Data Availability

Data is contained within the article and Supplementary Materials.

## Supplementary Materials

The following supporting information can be downloaded at: https://www.mdpi.com/article/10.3390/pharmaceutics15041285/s1. Table S1. Calibration curve data; Table S2. Statistical data related to the calibration curve (Excel); Figure S1. High-performance liquid chromatography (HPLC) chromatograms of (A) doxycycline standard, (B) doxycycline at pH 5.0, and (C) drug complex with sulfobutylether-β-cyclodextrin at pH 5.0. The concentration of all samples was 0.1 mg/mL; Table S3. High-performance liquid chromatography method performance data. Table S4. 1H NMR chemical shifts (δ) of doxycycline hyclate (DX), sulfobutyl-ether-β-cyclodextrin (SBE-β-CD), and the complexes obtained by freeze-drying; Figure S2. Nuclear magnetic resonance spectra of doxycycline (DX), sulfobutylether-β-cyclodextrin (SBE-β-CD), and the complex; Figure S3. Molecular structures of doxycycline and sulfobutylether-β-cyclodextrin; Table S5: Main vibrational modes of infrared spectra (4000–650 cm^−1^) of hyclate doxycycline (DX), sulfobutylether-β-cyclodextrin (SBE-β-CD), the physical mixture (1:4 molar ratio), and the freeze-dried complex (1:4 molar ratio). Reference [53] has cited in Supplementary Materials.
