# Supplementary material for: Supramolecular Arrangement of Doxycycline with Sulfobutylether-β-Cyclodextrin: Impact on Nanostructuration with Chitosan, Drug Degradation and Antimicrobial Potency
Source: Pharmaceutics. 2023 Apr 19;15(4):1285. doi: 10.3390/pharmaceutics15041285 (PMC10144562; doi:10.3390/pharmaceutics15041285)
Supplement: Supplementary file 1 [file pharmaceutics-15-01285-s001.zip › pharmaceutics-2255050-supplementary.pdf]

## SUPPLEMENTARY MATERIAL

# SUPRAMOLECULAR ARRANGEMENT OF DOXYCYCLINE WITH SULFOBUTYLETHER-β-CYCLODEXTRIN: IMPACT ON NANOSTRUCTURATION WITH CHITOSAN, DRUG DEGRADATION AND ANTIMICROBIAL POTENCY

Renata de Carvalho Feitosa [a], Juliana Souza Ribeiro Costa [a], Marcelo van Vliet Lima [a], Elina Sawa Akioka Ishikawa [a], Karina Cogo Müller [a], Fernando Bonin Okasaki [b], Edvaldo Sabadini [b], Claudia Garnero [c], Marcela Raquel Longhi [c], Vladimir Lavayen [d], Arnóbio Antônio da Silva-Júnior [e] and Laura Oliveira-Nascimento *[a].

[a] Faculty of Pharmaceutical Sciences, University of Campinas (UNICAMP), Campinas - SP, Brazil.

[b] Department of Physical Chemistry, Institute of Chemistry, University of Campinas (UNICAMP), Campinas - SP, Brazil.

[c] Research and Pharmaceutical Technology Development Unit (UNITEFA, CONICET-UNC) and Department of Pharmacy, Faculty of Chemical Sciences, National University of Córdoba, Córdoba, Argentina.

[d] Department of Inorganic Chemistry, Institute of Chemistry, Federal University of Rio Grande do Sul (UFRGS), Porto Alegre - RS, Brazil.

[e] Laboratory of Pharmaceutical Technology and Biotechnology, Department of Pharmacy, Federal University of Rio Grande do Norte (UFRN), Natal - RN, Brazil.

*Correspondence: Laura Oliveira-Nascimento (lauraon@unicamp.br)

HPLC Method performance data:

Linearity: linear range between 4.88 – 97.62 µg/mL ($r^2 = 0.9996$). The calibration curve data are in **Table S1** and the related statistical data are in **Table S2**.

Detection Limit (DL) and Quantification Limit (QL): DL and QL were calculated according to the following equations:

$$DL = \frac{3.3\sigma}{S}$$

$$QL = \frac{10\sigma}{S}$$

Where: S = slope of the calibration curve; σ = standard deviation of y-intercept of the regression line, as described by ICH [1]. The results are shown in **Table S3**.

Selectivity: complexation did not change Doxycycline retention time nor the major related substance peak in a significant manner (**Figure S1**). The filtrate of blank nanoparticles did not change the baseline.

Precision: repeatability (intra-day) analysis was carried out using three concentrations (low, medium and high) that contemplate the linear range of the analytical method, with three replicates at each level. The test was performed under the same operating conditions, same analyst, and same instrumentation, in a single analytical run. The relative standard deviation (RSD) was calculated, indicating the accuracy of the method. Intermediate precision (inter-day) was carried out under the same conditions of the previous analysis, but on a different day. The results are shown in **Table S3**.

Accuracy: The accuracy was inferred because linearity, precision and selectivity have been established (as recommended by ICH [1]). In addition, a drug recovery test was performed in the dispersing medium containing the empty nanoparticles. The results obtained indicate that the diluent and the matrix do not have significant interference in the quantification of the drug. The assay was performed in triplicate using an intermediate concentration of the calibration curve (30 µg/mL), results are in **Table S3**.

**Table S1.** Calibration curve data.

| *Drug concentration (µg/mL)* | *Peak area (mAU)* | *Average peak area (mAU)* | *RSD (%)* |
|---|---|---|---|
| 4.881 | 380373<br>396550<br>379676 | 385533 | 2.48 |
| 9.762 | 891925<br>930012<br>936594 | 919510 | 2.62 |
| 24.405 | 2563578<br>2505054<br>2546503 | 2538378 | 1.19 |
| 48.810 | 5281628<br>5447629<br>5406346 | 5378534 | 1.61 |
| 97.620 | 11206014<br>11263016<br>11307691 | 11258907 | 0.45 |

**Table S2.** Statistical data related to the calibration curve (excel)

| *Regression statistics* | |
|---|---|
| multiple R | 0.999808099 |
| R-squared | 0.999616235 |
| Adjusted R-squared | 0.999586715 |
| Standard error | 83772.98214 |
| Observation | 15 |

**ANOVA**

| | *df* | *SS* | *MS* | *F* | *Significance F* |
|---|---|---|---|---|---|
| Regression | 1 | 2.3764E+14 | 2.3764E+14 | 33861.92 | 1.35869E-23 |
| Residual | 13 | 91232862967 | 7017912536 | | |
| Total | 14 | 2.37731E+14 | | | |

| | *Coefficients* | *Standard error* | *t Stat* | *P-value* | *Lower 95%* | *Upper 95%* | *Lower 95.0%* | *Upper 95.0%* |
|---|---|---|---|---|---|---|---|---|
| Intercept | -259187.3129 | 32063.25649 | -8.0836241 | 2E-06 | -328455.7672 | -189918.8585 | -328455.7672 | -189918.8585 |
| Variable X 1 | 117409.0704 | 638.0370361 | 184.016074 | 1.36E-23 | 116030.6752 | 118787.4656 | 116030.6752 | 118787.4656 |

**Residue results**

| *Observation* | *Predicted Y* | *Residual* | *standardized residual* | *Observation* | *Predicted Y* | *Residual* | *standardized residual* |
|---|---|---|---|---|---|---|---|
| 1 | 313886.3599 | 66486.64013 | 0.823612165 | 8 | 2606181.051 | -101127.0509 | -1.252724895 |
| 2 | 313886.3599 | 82663.64013 | 1.024006921 | 9 | 2606181.051 | -59678.05086 | -0.739269853 |
| 3 | 313886.3599 | 65789.64013 | 0.814977984 | 10 | 5471549.415 | -189921.4146 | -2.352676976 |
| 4 | 886960.0326 | 4964.967385 | 0.061504199 | 11 | 5471549.415 | -23920.41459 | -0.296317342 |
| 5 | 886960.0326 | 43051.96739 | 0.533312016 | 12 | 5471549.415 | -65203.41459 | -0.807716037 |
| 6 | 886960.0326 | 49633.96739 | 0.614847423 | 13 | 11202286.14 | 3727.857933 | 0.04617934 |
| 7 | 2606181.051 | -42603.05086 | -0.527751002 | | | | |

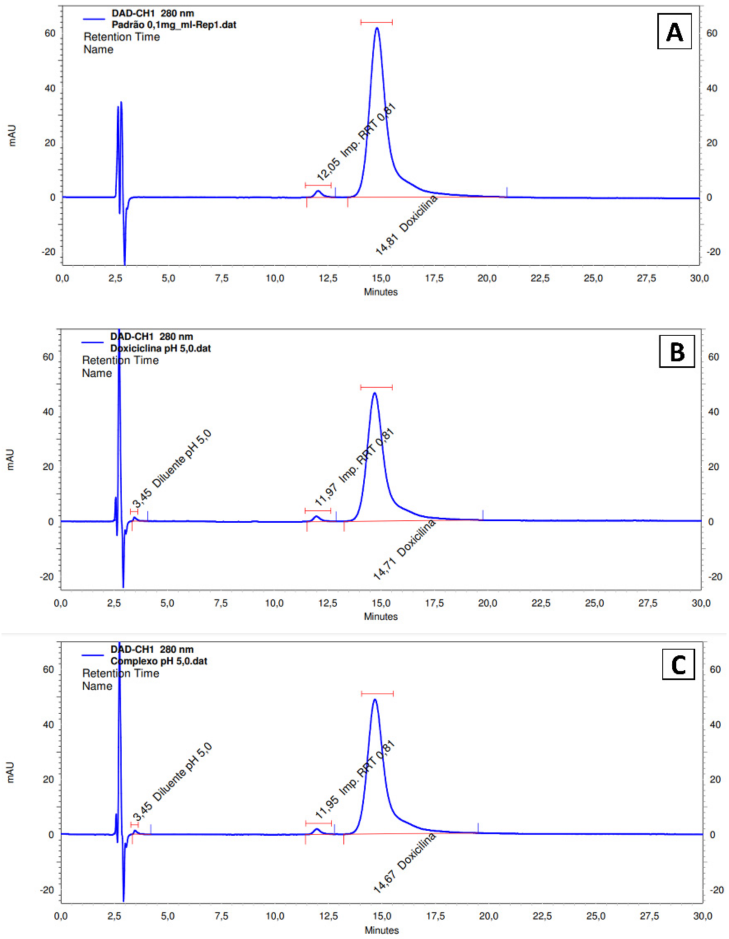


**Figure S1.** High-performance liquid chromatography (HPLC) chromatograms of **(A)** doxycycline standard, **(B)** doxycycline at pH 5.0 and (C) drug complex with sulfobutylether-β-cyclodextrin at pH 5.0. The concentration of all samples was 0.1 mg/mL.

**Table S3.** High Performance Liquid Chromatography method performance data.

| Parameter | Result |
|---|---|
| Selectivity | no interference detectable |
| Calibration Curve | y = 117409x - 259187 |
| Linearity (µg/mL) | 4.88 – 97.62 |
| Coefficient of Determination ($R^2$) | 0.9996 |
| DL (µg/mL) | 1.045 |
| QL (µg/mL) | 3.168 |
| Accuracy (% Recovery) * | 101.48 ± 2.52 |
| Precision intra-day (repeatability % RSD) | High level (4.9 µg/mL): 2.48<br>Medium level (24.4 µg/mL): 1.19<br>Low level (97.6 µg/mL): 0.45 |
| Precision inter-day (intermediate precision % RSD) | High level (5.2 µg/mL): 1.12<br>Medium level (25.8 µg/mL): 2.75<br>Low level (103.3 µg/mL): 0.80 |

DL: Detection Limit; QL: Quantification Limit. * Accuracy inferred by the recovery experiment.

**Table S4**. $^{1}$H NMR chemical shifts (δ) of doxycycline hyclate (DX), sulfobutyl-ether-β-cyclodextrin (SBE-β-CD), and the complexes obtained by freeze-drying.

| | Assignment | $\delta_{free}$ | Complex ($\Delta\delta = \delta_{system} - \delta_{free}$) |
|---|---|---|---|
| DX | H6 | 2.65 | 0.02 |
| | H7 | 6.76 | 0.29 |
| | H8 | 7.33 | 0.29 |
| | H9 | 6.56 | 0.28 |
| | $HCH_3$ | 1.38 | 0.22 |
| | $NH_2$ | 7.49 | No signal |
| SBE-β-CD | H1 | 5.23, 5.09 | -0.02, -0.01 |
| | H1' | 3.74 | -0.03 |
| | H2 | 3.62 | Overlap |
| | H3 | 4.05 | No signal |
| | H2' - H3' | 1.80 | -0.01 |
| | H4' | 2.97 | Overlap |
| | H5 | 3.89 | 0 |

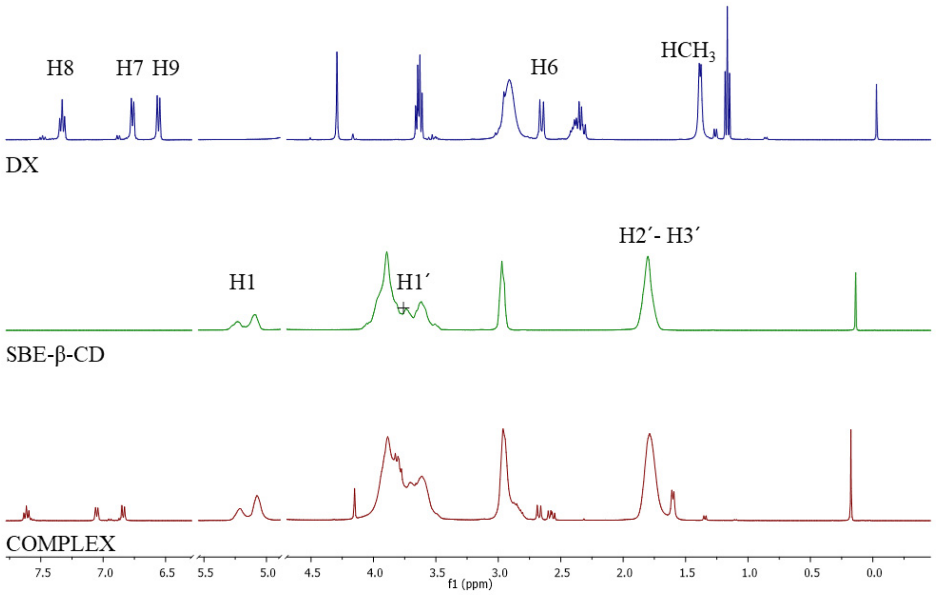


**Figure S2.** Nuclear magnetic resonance spectra of doxycycline (DX), sulfobutylether-β-cyclodextrin (SBE-β-CD) and complex.

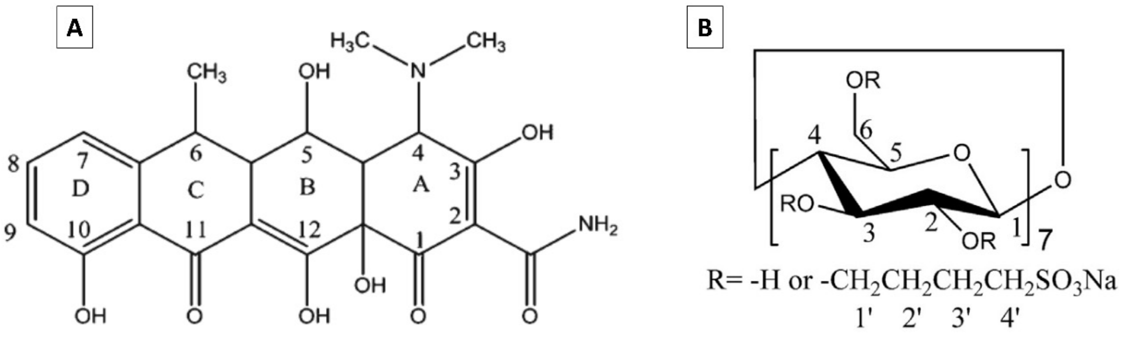


**Figure S3.** Molecular structure of doxycycline and sulfobutylether-β-cyclodextrin.

**Table S5**: Main vibrational modes of infrared spectra (4000 – 650 cm-1) of hyclate doxycycline (DX), sulfobutylether-β-cyclodextrin (SBE-β-CD), physical mixture (1:4 molar ratio), and freeze-dried complex (1:4 molar ratio).

| **DX ($cm^{-1}$)** | **SBE-β-CD ($cm^{-1}$)** | **Mixture ($cm^{-1}$)** | **Complex ($cm^{-1}$)** | **Assignment** |
|---|---|---|---|---|
| | 3619(66)* | 3607(51)* | | ν(O-H), CD partial Hbond |
| 3440(83)* | | | | |
| | 3548(109) | 3545(86) | 3556(128)* | ν(O-H), CD partial Hbond |
| | 3416(172) | 3428(157) | | ν(H-O-H), retained in the interstices among CD |
| | | | 3401(186) | ν(H-O-H) |
| 3372(42) | | | | ν(OH) |
| 3331(33) | | | | |
| 3285(179) | | 3287(81) | | ν(O-H), CD |
| | 3266(224) | | 3267(197) | |
| | | | 3227(114) | ν(O-H) |
| | | 3153(164) | | |
| $2934_{(w)}$ | | $2934_{(w)}$ | $2939_{(w)}$ | $\nu_{as}(CH_2)$ |
| $2880_{(vw)}$ | | $2880_{(vw)}$ | $2880_{(vw)}$ | $\nu_{s}(CH_2)$ |
| $1710_{(sh)}$ | | | | |
| | $1664_{(w)}$ | | | δ(H-O-H) bound to CD |
| $1643_{(m)}$ | | $1656_{(w,vw)}$ | $1651_{(vw)}$ | ν(C=O), DX |
| | $1610_{(m)}$ | | | ν(C=C), DX |
| | $1556_{(m)}$ | $1544_{(vw)}$ | | |
| $1452_{(m,vw)}$ | $1456_{(m)}$ | $1453_{(m,vw)}$ | $1451_{(vw)}$ | φOCH, φHCH |
| | | $1422_{(m,vw)}$ | $1420_{(vw)}$ | φOCH, φCCH |
| $1415_{(m,vw)}$ | | $1411_{(m,vw)}$ | | |
| $1366_{(m,vw)}$ | $1362_{(m)}$ | $1364_{(m,vw)}$ | $1365_{(vw)}$ | φCCH, φOCH, φCOH δ(C-H), CD/in plane bending of CH, enolic COH, skeletal CCC |
| $1330_{(vw)}$ | $1330_{(m)}$ | | | Complex modes $CH_2OH$, CD |
| | $1301_{(m)}$ | | | ν(S=O) |
| $1261_{(vw)}$ | | | | $\delta_{in\text{-}plane}$(C-H) |
| | $1240_{(sh,m)}$ | | | Complex modes CH2OH, CD |
| | $1222_{(m)}$ | | | |
| $1196_{(sh,m)}$ | | $1202_{(sh,m)}$ | $1198_{(sh,m)}$ | |
| | $1171_{(sh,m)}$ | | | |
| $1153_{(s)}$ | | $1156_{(m)}$ | $1156_{(m)}$ | νCO,νCC ν(C-O-C) glucosidic/CH overtone stretching |
| | $1125_{(w)}$ | | | ν(S=O) |
| | $1041_{(w)}$ | | | ν(C-C) |
| 1044 | | | | ν(C-N)/DX |
| $1027_{(m)}$ | | $1029_{(w)}$ | $1029_{(w)}$ | $\delta_{in\text{-}plane}$(C-H) DX /νCC, δOCH, δCCH, δCCO |
| | $1026_{(w)}$ | | | νCC, δOCH, δCCH, δCCO |
| $946_{(vw)}$ | | | | |
| | $934_{(vw)}$ | | | Skeletal vibration a-1,4 linkage |

| | |
|---|---|
| $858_{(vw)}$ | |
| $788_{(vw)}$ | δ *Out-of-plane* (=C-H) DX |

[a] Relative intensity: w = weak; s = strong; sh = shoulder; m = medium; vw = very weak; υ = stretching mode; δ = bending mode; mm = multiple; φ = stretching mode guest ring. The values among brackets are the FWHM numbers to come from deconvolution process on the complex 1:4.

References:

[1] International Council for Harmonisation. ICH Harmonised Guideline: Validation of analytical procedures Q2(R2). Available online: https://www.ich.org/page/quality-guidelines (accessed on 30 March 2023).
